# Supplementary material for: Quantifying antibiotic impact on within-patient dynamics of extended-spectrum beta-lactamase resistance
Source: eLife. 2020 May 7;9:e49206. doi: 10.7554/eLife.49206 (PMC7205461; doi:10.7554/eLife.49206)
Supplement: Reporting standard 1. [file elife-49206-repstand1.pdf]

Niehus et al. – STROBE guidelines [Von Elm et al., 2007]

Item	Number	Recommendation	Addressed where
Title and abstract	1a	Indicate the study's design with a commonly used term in the title or the abstract	"prospective cohort study" (**Abstract**)
	1b	Provide in the abstract an informative and balanced summary of what was done and what was found	see Abstract
Introduction			
Background/rationale	2	Explain the scientific background and rationale for the investigation being reported	see Introduction
Objectives	3	State specific objectives, including any prespecified hypotheses	see Introduction
Methods			
Study design	4	Present key elements of study design early in the paper	see "Patient cohort and treatment" and Table 1 (**Results**)
Setting	5	Describe the setting, locations, and relevant dates, including periods of recruitment, exposure, follow-up, and data collection	see "Patient cohort and treatment" and Table 1 (**Results**)
Participants	6a	Give the eligibility criteria, and the sources and methods of selection of participants. Describe methods of follow-up	for eligibility criteria see Table 1, for follow-up methods see "Study participants and follow-up" (**Methods and Materials**)
	6b	For matched studies, give matching criteria and number of exposed and unexposed	Not applicable
Variables	7	Clearly define all outcomes, exposures, predictors, potential confounders, and effect modifiers. Give diagnostic criteria, if applicable	Outcome and predictors explained in **Methods and Materials**. Distribution of patients' hospital origin, age & sex described in "Patient cohort and treatment" (**Results**)

			Diagnostic method of ESBL colonisation in "Identification of ESBL producing organisms carriers" (**Methods and Materials**)
Data sources/measurement	8	For each variable of interest, give sources of data and details of methods of assessment (measurement). Describe comparability of assessment methods if there is more than one group	"Study participants and follow-up" (**Methods and Materials**) explains source of antibiotic treatment data, and microbiological methods are given the two sections following
Bias	9	Describe any efforts to address potential sources of bias	Selection bias discussed in **Discussion and Conclusion**
Study size	10	Explain how the study size was arrived at	Explained in "Study participants and follow-up" (**Methods and Materials**)
Quantitative variables	11	Explain how quantitative variables were handled in the analyses. If applicable, describe which groupings were chosen and why	see **Methods and Materials** sections of the statistical methods
Statistical methods	12	a) Describe all statistical methods, including those used to control for confounding<br>b) Describe any methods used to examine subgroups and interactions<br>c) Explain how missing data were addressed<br>d) If applicable, explain how loss to follow-up was addressed<br>e) Describe any sensitivity analyses	Statistical methods described and justified in **Methods and Materials**. Potential for interactions between antibiotics discussed in **Discussion and Conclusion**. No missing data and no loss to follow-up. We describe model comparison in "Dynamic within-host model" (**Methods and Materials**)
Results			

Participants	13a	Report numbers of individuals at each stage of study—eg numbers potentially eligible, examined for eligibility, confirmed eligible, included in the study, completing follow-up, and analysed	Number of total patients and patients consenting to follow-up given in “Patient cohort and treatment” (**Results**)
	13b	Give reasons for non-participation at each stage	Reasons unknown
	13c	Consider use of a flow diagram	Deemed not necessary
Descriptive data	14a	Give characteristics of study participants (eg demographic, clinical, social) and information on exposures and potential confounders	Distribution of patients’ hospital origin, age and sex given in “Patient cohort and treatment” (**Results**)
	14b	Indicate number of participants with missing data for each variable of interest	no missing data
	14c	Summarise follow-up time (eg, average and total amount)	Median and maximum length of observation, and total number of observed days (with and without antibiotic treatment) given in “Patient cohort and treatment” (**Results**)
Outcome data	15	Report numbers of outcome events or summary measures over time	Raw data in Figure 1, and summaries in Figure 2 and 3 (with and without treatment)
Main results	16a	Give unadjusted estimates and, if applicable, confounder-adjusted estimates and their precision (eg, 95% confidence interval). Make clear which confounders were adjusted for and why they were included	Estimates given visually (Figure 4 + 5) and in text in “Dynamic antibiotic effect model” (**Results**)
	16b	Report category boundaries when continuous variables were categorized	not applicable

	16c	If relevant, consider translating estimates of relative risk into absolute risk for a meaningful time period	not applicable
Other analysis	17	Report other analyses done—eg analyses of subgroups and interactions, and sensitivity analyses	Model comparison reported in "Dynamic antibiotic effect model" (**Results**)
Discussion			
Key results	18	Summarise key results with reference to study objectives	see **Discussion and Conclusion**
Limitations	19	Discuss limitations of the study, taking into account sources of potential bias or imprecision. Discuss both direction and magnitude of any potential bias	Limitation and potential bias discussed (**Discussion and Conclusion**)
Interpretation	20	Give a cautious overall interpretation of results considering objectives, limitations, multiplicity of analyses, results from similar studies, and other relevant evidence	see **Discussion and Conclusion**
Generalisability	21	Discuss the generalisability (external validity) of the study results	see **Discussion and Conclusion**
Other information			
Funding	22	Give the source of funding and the role of the funders for the present study and, if applicable, for the original study on which the present article is based	Study funding given in Table 1 and in "Acknowledgements"
