# Supplementary material for: Quantifying antibiotic impact on within-patient dynamics of extended-spectrum beta-lactamase resistance
Source: eLife. 2020 May 7;9:e49206. doi: 10.7554/eLife.49206 (PMC7205461; doi:10.7554/eLife.49206)
Supplement: Reporting standard 2. [file elife-49206-repstand2.pdf]

Niehus et al. – STROBE-AMS guidelines [Tacconelli *et al. BMJ Open* 2016]

Item	Number	STROBE-AMS Recommendation	Addressed where
Introduction	2	Report previous clinical in vivo and in vitro studies	Reported in **Introduction** and **Discussion and Conclusion**
Objectives	3	State specific objectives, including any prespecified hypotheses	See **Introduction**
Methods			
Setting	5.1	Describe if setting is epidemic or endemic (high, low, medium) for the study outcome	High prevalence setting of ESBL-producing bacterial carriage ("Study participants and follow-up", **Methods and Materials**)
	5.2	Specify type of hospital or unit and characteristics of population served by the healthcare setting	Medical and surgical wards, inpatients ("Study participants and follow-up", **Methods and Materials**)
	5.3	Describe antimicrobial formulary in use at the study location related to the analysed antibiotics	Ten most used antibiotics shown in Table 2, total number of antibiotics used, routes of administration and common antibiotioc classes described in text ("Patient cohort and treatment", **Results**)
	5.4	Describe infection control measures dedicated to the target resistant bacteria applied at the study location	No specific infection control measures targeting ESBL producing bacteria
Participants	6a	Cohort study—Give the eligibility criteria, the sources and methods of selection of participants. Describe methods of follow-up	see **Methods and Materials** under "Study participants and follow-up"
	6.1	Define unit analysed (person, department or other)	2 wards from 3 hospitals (high ESBL

			prevalence) representing within hospital population in similar settings ("Study participants and follow-up" in **Methods and Materials**). Data was analysed per patient. No sub-analysis per ward due to limited number of patients
	6.2	Provide reasons (epidemiological and clinical) for choosing matching criteria	Not applicable
Variables	7.1	Specify antimicrobial usage according to: type, dosage, duration and route of administration	Antibiotic treatment was analysed by type, route and accounting for duration ("Dynamic within-host model", **Methods and Materials**). Dosage was not considered, see discussion of pharmacodynamic modelling (**Discussion and Conclusion**)
	7.2	Provide information using defined daily dosages (DDDs) and, in addition, other definitions closer to local reality (packages, prescriptions). Provide justification for the measurement presented	see 7.1
	7.3	Address antimicrobial combinations	Multivariate dynamic model accounts for combinations. See also discussion of multiplicative effects (**Discussion and Conclusion**)
	7.4	Explain rationale for grouping of antimicrobials	Grouping explained in "Association of antibiotic treatment

			and changes in resistance" (**Methods and Materials**). Antibiotics were considered individually in dynamic modelling ("Dynamic within-host model", **Methods and Materials**)
	7.5	Define time at risk for antimicrobial exposure and for resistance development	Study considered only within hospital treatment and pre-existing ESBL resistance
	7.6	Include description of potential confounders (other than epidemiological variables)	Distribution of patients' hospital origin, age and sex give ("Patient cohort and treatment", **Results**)
	7.7	Provide definition of resistance, multidrug resistance, including pattern of coresistance; whether studies performed to identify location or resistance eg, plasmid, chromosome, integron, transposon	ESBL resistance was identified phenotypically ("Identification of ESBL producing organism carriers", **Methods and Materials**) and detected genetically as bla_{CTX-M} abundance. Co-resistance patters were not established
	7.8	Definition of infection and/or colonisation. If not a validated reference, provide evidence of robustness of the new definition	Colonisation was identified as described in "Identification of ESBL producing organism carriers" (**Methods and Materials**). Only carriage, not infections were considered

Data sources/measurement	8.1	8.1 Describe how antimicrobial consumption data were obtained (pharmacy, patients' charts, etc) and if it was actually used or purchased/dispensed	Antibiotic treatment data from patients' charts ("Study participants and follow-up", **Methods and Materials**)
Quantitative variables	11.1	Provide subgroup analyses for immunocompromised, surgical/medical patients and patients in intensive care units, if applicable	Stratification by ward (medical vs surgical) was not feasible due to limited number of patients
Results			
Descriptive data	14.1	Specify among the exposure: previous stay in long-term care facilities, nursing home and other healthcare settings	No on prior hospitalisation available
Other analysis	17.1	Report subgroup analysis by type of patients and type of microorganism, if applicable	see 11.1
Discussion			
Limitations	19.1	Provide description of sources of selection bias, including infection control measures, audit and confounding	Discussed in **Discussion and Conclusion**
Generalisability	21.1	Discuss study setting, type of hospital, local epidemiology for the generalisability	Discussed in **Discussion and Conclusion**
