# Supplementary material for: Quantifying antibiotic impact on within-patient dynamics of extended-spectrum beta-lactamase resistance
Source: eLife. 2020 May 7;9:e49206. doi: 10.7554/eLife.49206 (PMC7205461; doi:10.7554/eLife.49206)
Supplement: Transparent reporting form [file elife-49206-transrepform.pdf]

eLIFE
1st Floor
24 Hills Road
Cambridge CB2 1JP, UK
P 01223 855340
W elifesciences.org
T @elife

eLife's transparent reporting form

We encourage authors to provide detailed information *within their submission* to facilitate the interpretation and replication of experiments. Authors can upload supporting documentation to indicate the use of appropriate reporting guidelines for health-related research (see EQUATOR Network), life science research (see the BioSharing Information Resource), or the ARRIVE guidelines for reporting work involving animal research. Where applicable, authors should refer to any relevant reporting standards documents in this form.

If you have any questions, please consult our Journal Policies and/or contact us: editorial@elifesciences.org.

Sample-size estimation

- You should state whether an appropriate sample size was computed when the study was being designed
- You should state the statistical method of sample size computation and any required assumptions
- If no explicit power analysis was used, you should describe how you decided what sample (replicate) size (number) to use

Please outline where this information can be found within the submission (e.g., sections or figure legends), or explain why this information doesn't apply to your submission:

In the section "Study Participants and follow-up" (**Methods and Materials**) we explain the sample size calculations

Replicates

- You should report how often each experiment was performed
- You should include a definition of biological versus technical replication
- The data obtained should be provided and sufficient information should be provided to indicate the number of independent biological and/or technical replicates
- If you encountered any outliers, you should describe how these were handled
- Criteria for exclusion/inclusion of data should be clearly stated
- High-throughput sequence data should be uploaded before submission, with a private link for reviewers provided (these are available from both GEO and ArrayExpress)

Please outline where this information can be found within the submission (e.g., sections or figure legends), or explain why this information doesn't apply to your submission:

eLIFE
1st Floor
24 Hills Road
Cambridge CB2 1JP, UK
P 01223 855340
W elifesciences.org
T @elife

We explain in the “Quantitative PCR” section (**Methods and Materials**) that the qPCR measurement repeats served as technical replication. Because this is an observational study, there are no biological replicates. We show raw data of bla_{CTX-M} abundance in Figure 1 and provide the full dataset online (https://datadryad.org/review?doi=doi:10.5061/dryad.8vf034). There were no missing values and we did not exclude outliers, but instead accounted for differential variation of qPCR measurement (“Dynamic within-host model” **Methods and Materials**).

In our reporting, we also follow the STROBE guidelines for cohort studies [Von Elm et al., 2007] as well as the STROBE-AMS guidelines [Tacconelli et al., 2016], and the MICRO guidelines for reporting and interpretation of clinical microbiology data [Turner et al., 2019].

eLife Sciences Publications, Ltd is a limited liability non-profit non-stock corporation incorporated in the State of Delaware, USA, with company number 5030732, and is registered in the UK with company number FC030576 and branch number BR015634 at the address 1st Floor, 24 Hills Road, Cambridge CB2 1JP | August 2014

Statistical reporting

- Statistical analysis methods should be described and justified
- Raw data should be presented in figures whenever informative to do so (typically when N per group is less than 10)
- For each experiment, you should identify the statistical tests used, exact values of N, definitions of center, methods of multiple test correction, and dispersion and precision measures (e.g., mean, median, SD, SEM, confidence intervals; and, for the major substantive results, a measure of effect size (e.g., Pearson's r, Cohen's d)
- Report exact p-values wherever possible alongside the summary statistics and 95% confidence intervals. These should be reported for all key questions and not only when the p-value is less than 0.05.

Please outline where this information can be found within the submission (e.g., sections or figure legends), or explain why this information doesn't apply to your submission:

We describe each statistical method and its justification in their respective sections in **Methods and Materials**. We visualize the raw bla_{CTX-M} values in Figure 1, demonstrating the large variability in dynamical patterns. In the **Results** section "Associating resistance and antibiotic treatment" we give the numbers of contrasting intervals for each of the three comparisons, and we give the numbers of the patients, swabs and qPCR measurements used in the dynamic model in "Dynamic antibiotic effect model". We report the priors used for the Bayesian models in the respective section in Methods and Materials. Instead of giving p-values, we summarise posterior distributions visually (Figure 4) and using summary statistics ("Dynamic antibiotic effect model" in **Results**).

(For large datasets, or papers with a very large number of statistical tests, you may upload a single table file with tests, Ns, etc., with reference to sections in the manuscript.)

Group allocation

- Indicate how samples were allocated into experimental groups (in the case of clinical studies, please specify allocation to treatment method); if randomization was used, please also state if restricted randomization was applied
- Indicate if masking was used during group allocation, data collection and/or data analysis

Please outline where this information can be found within the submission (e.g., sections or figure legends), or explain why this information doesn't apply to your submission:

This was an observational study, and as such, patient treatment happened at discretion of the treating clinician.

Additional data files ("source data")

- We encourage you to upload relevant additional data files, such as numerical data that are represented as a graph in a figure, or as a summary table
- Where provided, these should be in the most useful format, and they can be uploaded as "Source data" files linked to a main figure or table
- Include model definition files including the full list of parameters used
- Include code used for data analysis (e.g., R, MatLab)
- Avoid stating that data files are "available upon request"

eLife Sciences Publications, Ltd is a limited liability non-profit non-stock corporation incorporated in the State of Delaware, USA, with company number 5030732, and is registered in the UK with company number FC030576 and branch number BR015634 at the address 1st Floor, 24 Hills Road, Cambridge CB2 1JP | August 2014

Please indicate the figures or tables for which source data files have been provided:

The Bayesian model code in Stan, the R code to fit the model, and the data in Rdata format as well as csv format are deposited in Dryad under https://datadryad.org/review?doi=doi:10.5061/dryad.8vf034t.

eLife Sciences Publications, Ltd is a limited liability non-profit non-stock corporation incorporated in the State of Delaware, USA, with company number 5030732, and is registered in the UK with company number FC030576 and branch number BR015634 at the address 1st Floor, 24 Hills Road, Cambridge CB2 1JP | August 2014
